# Halofuginone prevents outer retinal degeneration in a mouse model of light-induced retinopathy

**DOI:** 10.1371/journal.pone.0300045

**Published:** 2024-03-27

**Authors:** Yukihiro Miwa, Deokho Lee, Chiho Shoda, Heonuk Jeong, Kazuno Negishi, Toshihide Kurihara

**Affiliations:** 1 Laboratory of Photobiology, Keio University School of Medicine, Tokyo, Japan; 2 Department of Ophthalmology, Keio University School of Medicine, Tokyo, Japan; 3 Aichi Animal Eye Clinic, Aichi, Japan; 4 Department of Ophthalmology, Nihon University School of Medicine, Tokyo, Japan; Universidade Federal do ABC, BRAZIL

## Abstract

Photoreceptor cell death can cause progressive and irreversible visual impairments. Still, effective therapies on retinal neuroprotection are not available. Hypoxia-inducible factors (HIFs) are transcriptional factors which strongly regulate angiogenesis, erythropoiesis, intracellular metabolism, and programed cell death under a hypoxic or an abnormal metabolic oxidative stress condition. Therefore, we aimed to unravel that inhibition of HIFs could prevent disease progression in photoreceptor cell death, as recent studies showed that HIFs might be pathologic factors in retinal diseases. Adult male balb/cAJcl (8 weeks old; BALB/c) were used to investigate preventive effects of a novel HIF inhibitor halofuginone (HF) on a murine model of light-induced retinopathy. After intraperitoneal injections of phosphate-buffered saline (PBS) or HF (0.4 mg/kg in PBS) for 5 days, male BALB/c mice were subjected to a dark-adaption to being exposed to a white LED light source at an intensity of 3,000 lux for 1 hour in order to induce light-induced retinal damage. After extensive light exposure, retinal damage was evaluated using electroretinography (ERG), optical coherence tomography (OCT), and TUNEL assay. Light-induced retinal dysfunction was suppressed by HF administration. The amplitudes of scotopic a-wave and b-wave as well as that of photopic b-wave were preserved in the HF-administered retina. Outer retinal thinning after extensive light exposure was suppressed by HF administration. Based on the TUNEL assay, cell death in the outer retina was seen after light exposure. However, its cell death was not detected in the HF-administered retina. Halofuginone was found to exert preventive effects on light-induced outer retinal cell death.

## Introduction

Age-related macular degeneration (AMD), a multifactorial and progressive degenerative disease, has been known to be the main cause of visual loss in many industrialized countries with aging. As reactive oxygen species could be accumulated in the retina, excessive light exposure is one of the pathologic risk factors to induce degeneration of photoreceptors under the AMD condition [1, 2]. In that reason, research scientists have focused on understanding the mechanism of light-induced retinopathy [3, 4]. To understand the pathologic mechanisms for photoreceptor damage, a murine light-induced retinopathy (LIR) model has been widely used [5–7]. Furthermore, the LIR model was applied to screen promising drug candidates in this aspect. A number of preclinical studies suggested that early treatment or pre-treatment of therapeutic agents to suppress oxidative stress in the retina could reduce photoreceptor damage [8–12]. However, promising therapeutic molecular targets remain unclear.

Hypoxia-inducible factors (HIFs) are transcriptional factors that strongly regulate hypoxia-responsive or metabolic stress-responsive genes against hypoxic or non-hypoxic oxidative stress [13–16]. While HIFs are rapidly degraded by the ubiquitin proteasome pathway with hydroxylation via prolyl hydroxylases (PHDs) under normoxia, HIFs can be stabilized with reductions of hydroxylation under hypoxia [13, 17]. Then, stabilized HIFs finally lead to the activation of HIF target gene expressions, including vascular endothelial cell growth factor (VEGF), BCL2/adenovirus E1B 19 kDa protein-interacting protein 3 (BNIP3), adrenomedulin (ADM), angiopoietin-like 2 and 4 (ANGPTL2 and ANGPTL4), endothelin-1 (ET-1), heme oxygenease-1 (HO-1), nitric oxide synthase (NOS), or stromal derived growth factor-1 (SDF-1) [18–20]. Depending on the tissue condition, those gene expressions might be pathologic or protective to the retina. Oxidative stress could also increase HIF expression in ocular tissues, finally leading to the upregulation of various HIF target gene expressions [21]. Increased expressions in hypoxia-related genes were detectible in the aged human retina, which implies that photoreceptor cells might be related to the upregulation of hypoxia-induced HIF target genes in response to a mild but chronic hypoxic stress that may occur under aging process [22]. Many groups previously suggested that HIFs may be involved in retinal diseases and that the inhibition of HIFs may have therapeutic effects in various mouse models of neurodegenerative diseases [18, 22–25]. However, its effects have not been clearly examined in a murine model of LIR.

Halofuginone is an analog of febrifugine (an alkaloid originally isolated from *Dichroa febrifugea*). Halofuginone recently received much attention for its wide range of biological benefits in malaria, cancer, and fibrosis-associated immune diseases [26]. Anti-angiogenic effects of halofuginone were examined in an in vivo model of acute promyelocytic leukemia [27]. Furthermore, PCR analysis of bone marrow samples showed reductions in VEGF and HIF-1 gene expressions in halofuginone-treated mice [27]. Halofuginone has been reported to strongly suppress HIF expressions in the retina in vivo and in a retinal cell line [28, 29]. In that HIF inhibitory effects of halofuginone were examined in a mouse model of retinal ischemia/reperfusion injury model [28, 29], its HIF inhibitory activity has not been examined in the LIR model. Therefore, in this study, we aimed to investigate whether a HIF inhibitor halofuginone could prevent outer retinal degeneration in a murine model of LIR.

## Materials and methods

### Animals

Animals were purchased from CLEA Japan (Tokyo, Japan), and maintained in a temperature and cycle-controlled environment (24 ± 1°C; 12 h light-12 h dark). Supplies, such as food and water, were freely given to animals. Animal experiments were approved by the Ethics Committee on Animal Research of the Keio University School of Medicine (#16017). The ARVO Statement for the Use of Animals in Ophthalmic and Vision Research, and the international standards of animal care and use, Animal Research: Reporting in vivo Experiments guidelines were also followed in our current study.

During experiments in which signs including hunched posture, lethargy, lack of food intake or unexpected infection were detected in mice, a combination of 3x of medetomidine (7.5 μg/100 μL; Orion, Espoo, Finland), midazolam (40 μg/100 μL; Sandoz, Tokyo, Japan), and butorphanol tartrate (50 μg/100 μL; Meiji Seika Pharma, Tokyo, Japan) “MMB” was intraperitoneally administered to mice. Then, under deep anesthesia, mice were euthanized. Alleviation of suffering was not required, as there was no surgical operation to induce pain in mice in our experimental scheme.

### Light-induced retinopathy

Male balb/cAJcl (8 weeks old; BALB/c) mice were subjected to a dark-adaption for at least 12 hours. The BALB/c mouse is not only generally and widely used in vision research, but also its mouse line was reported more susceptible to light damage in comparison to C57BL/6 mice [30–32]. Based on our previous research, we also found that BALB/c mice were susceptible to light damage in our system [33]. After dark-adaption, each mouse was placed in each cage and exposed to a white LED light source at an intensity of 3,000 lux in order to cause damage in the outer retina, as previously described [33]. After one hour of extensive light exposure, mice were back to their original cages. This light exposure was conducted in room temperature.

### Halofuginone administration

BALB/c mice were randomly allocated into two experimental groups: one group received halofuginone (Cat number: 32481, Sigma; 0.4 mg/kg/day in phosphate-buffered saline [PBS]) via intraperitoneal injection, while the other group was provided with an equivalent volume of PBS as the HF’s control. Five consecutive injections of HF or PBS were performed before extensive light exposure at an intensity of 3,000 lux. Each fresh frozen stock of halofuginone was thawed on the day of use. The dosage was determined approximately based on the several previous HF literatures [28, 34–36]. The HF or PBS injection was at 24-hour intervals. On the day of light exposure, the injection was performed before 1 hour of light exposure.

### Electroretinography (ERG) and optical coherence tomography (OCT)

Seven days after light exposure, ERG and OCT were conducted, as previously described [37]. For ERG, after a dark-adaption for more than 12 hours, a mixture of MMB was injected to mice for anesthesia with pupil dilation. After anesthesia, mice were placed inside a Ganzfeld dome and scotopic and photopic ERG waves were recorded using a PuREC ERG acquisition machine (MAYO, Inazawa, Japan). Light-emitting diode (LED) stimulators were used for light source. Standardized light intensities (scotopic: 0.5, 2, 10, 50 cd.s/m^2^; photopic: 4, 10, 20 cd.s/m^2^) were applied to obtain the amplitudes of scotopic a-wave and b-wave and photopic b-wave in mice. The active electrodes were placed on the eyes. A reference electrode was inserted to the mouth. A clipping ground electrode was placed to the tail. ERG responses were obtained from both eyes. All mice were kept warm during the ERG procedure by using heat pads. The amplitude of a-wave was determined from the baseline to the lowest point of a-wave and the amplitude of b-wave was set from the lowest point of a-wave to the peak of b-wave. Amplitudes of scotopic a-wave and b-wave and photopic b-wave are represented in absolute values.

For OCT, after mouse anesthesia with pupil dilation, mice were softly fixed on an OCT platform. After mice were fixed on the platform, retinal images at 200, 400, 600, and 800 μm from the optic nerve head in the superior and inferior areas were taken by the Envisu R4310 OCT camera (Leica, Wetzlar, Germany). Outer retinal thickness was obtained by general software provided from Envisu R4310 OCT.

### TUNEL staining

TUNEL staining was performed as previously described [37]. After MMB injection, mice were euthanized. Eyeballs from mice were obtained and incubated in 4% paraformaldehyde (PFA) solution. The eyeballs fixed in 4% PFA solution were incubated in 30% sucrose solution and then subjected to sagittal-section by a general cryostat (CM3050S, Leica, Wetzlar, Germany). Then, the sectioned samples were subjected to TUNEL staining. We followed the general manufacturer’s instruction (written in the in situ Apoptosis Detection Kit, Cat #MK500, Takara Bio, Japan). After permeabilization, a reaction mixture containing TdT enzymes was applied to the sectioned samples. After 1 minute of 4’,6-diamidino-2-phenylindole (DAPI) incubation to the samples, the sectioned slides were mounted and examined via the LSM710 microscope (Carl Zeiss, Jena, Germany).

### Western blot

For western blot, the samples of retinas or 661W cells were homogenized in lysis RIPA buffer (Thermo Fisher Scientific, Waltham MA, USA) containing protease inhibitor cocktails (Roche, Switzerland). After protein lysates were boiled, the protein concentration of the lysates was determined by a bicinchoninic acid assay kit (Pierce, Rockford, IL, USA). The samples were fractionated on 10% SDS-polyacrylamide gels, transferred to nitrocellulose membranes, and blocked with 5% nonfat dry milk. Antibodies used in this study are as follows: anti-HIF-1α (1:1000, Cat #36169, Cell Signaling Technology, Danvers, MA, USA), anti-BNIP3 (1:2000, ab109362, Abcam, England), and anti-β-ACTIN (1:5000, #3700, Cell Signaling Technology, MA, USA). After incubation with primary antibodies in a 4-degree freezer overnight, membranes were washed with tris-buffered saline with 0.1% Tween (TBST) three times. Then, membranes were incubated with horseradish peroxidase-conjugated secondary antibody solutions (1:5000, GE Healthcare, Princeton, NJ, USA) for 2 hours at room temperature. After TBST washing three times, signals were detected using an ECL kit (Ez WestLumi plus, ATTO, Tokyo, Japan). Protein bands were visualized using chemiluminescence (ImageQuant LAS 4000 mini, GE Healthcare, Chicago, IL, USA) and quantified using NIH ImageJ software (National Institutes of Health, Bethesda, MD, USA).

### Quantitative PCR

For quantitative PCR, total RNA samples were extracted using a commercial kit (the RNeasy Plus Mini Kit, Qiagen, Venlo, The Netherlands), and its concentration was quantified using a spectrophotometer (NanoDrop 2000c, Thermo Scientific, Waltham, MA, USA), as previously described [38]. Then, total RNA samples were converted to cDNA samples using a commercial Kit (the ReverTra Ace qPCR RT Master Mix with gDNA Remover, TOYOBO, Osaka, Japan). Quantitative PCR was performed with a SYBR green master mix commercial kit (THUNDERBIRD SYBR qPCR Mix, TOYOBO, Osaka, Japan) using a PCR system (the Step One Plus Real-Time PCR system, Applied Biosystems, Waltham, MA, USA). The fold difference in different transcripts was calculated by the ΔΔCt protocol [39]. *Hprt* was used as a reference gene. Primers used in our current study as follows: *Hprt* (Forward: TCAGTCAACGGGGGACATAAA; Reverse: GGGGCTGTACTGCTTAACCAG), *Hif-1α* (Forward: GGTTCCAGCAGACCCAGTTA; Reverse: AGGCTCCTTGGATGAGCTTT), *Bnip3* (Forward: GCTCCCAGACACCACAAGAT; Reverse: TGAGAGTAGCTGTGCGCTTC). The retinal samples were prepared 3, 6, 9, 12, and 24 hours after light exposure.

### Luciferase assay

Luciferase assay was conducted, as previously indicated [38]. A murine cone photoreceptor cell line (661W cells) was transfected with the HIF-luciferase reporter gene construct (Cignal Lenti HIF Reporter, Qiagen, Venlo, Netherlands) to monitor the HIF transcriptional activity. The HIF-luciferase construct encodes the firefly luciferase gene under the control of hypoxia response element which closely binds to HIFs. These cells were co-transfected with the CMV-renilla luciferase construct as an internal control for general cellular conditions. Cobalt chloride (CoCl_2_, 200 μM, cobalt (II) chloride hexahydrate, Wako, Japan) was treated to the cells in order to induce HIF activation for 24 hours before measuring the luminescence. To evaluate suppressive effects of HF on HIF activation, HF was added to the cells at the same time as CoCl_2_ was added to the cells. The luminescence was measured with the Dual-Luciferase Reporter Assay System (Promega, Fitchburg, WI, USA).

### Statistical analysis

All data’ values were depicted as mean ± standard deviation. Statistical significance was determined by using a two-way Student’s *t*-test or a one- or two-way ANOVA followed by a Bonferroni post hoc test depending on the presented data, and P < 0.05 was considered statistically significant.

## Results

### HIF-1α expression increases in a murine model of light-induced retinopathy

**Fig 1:**
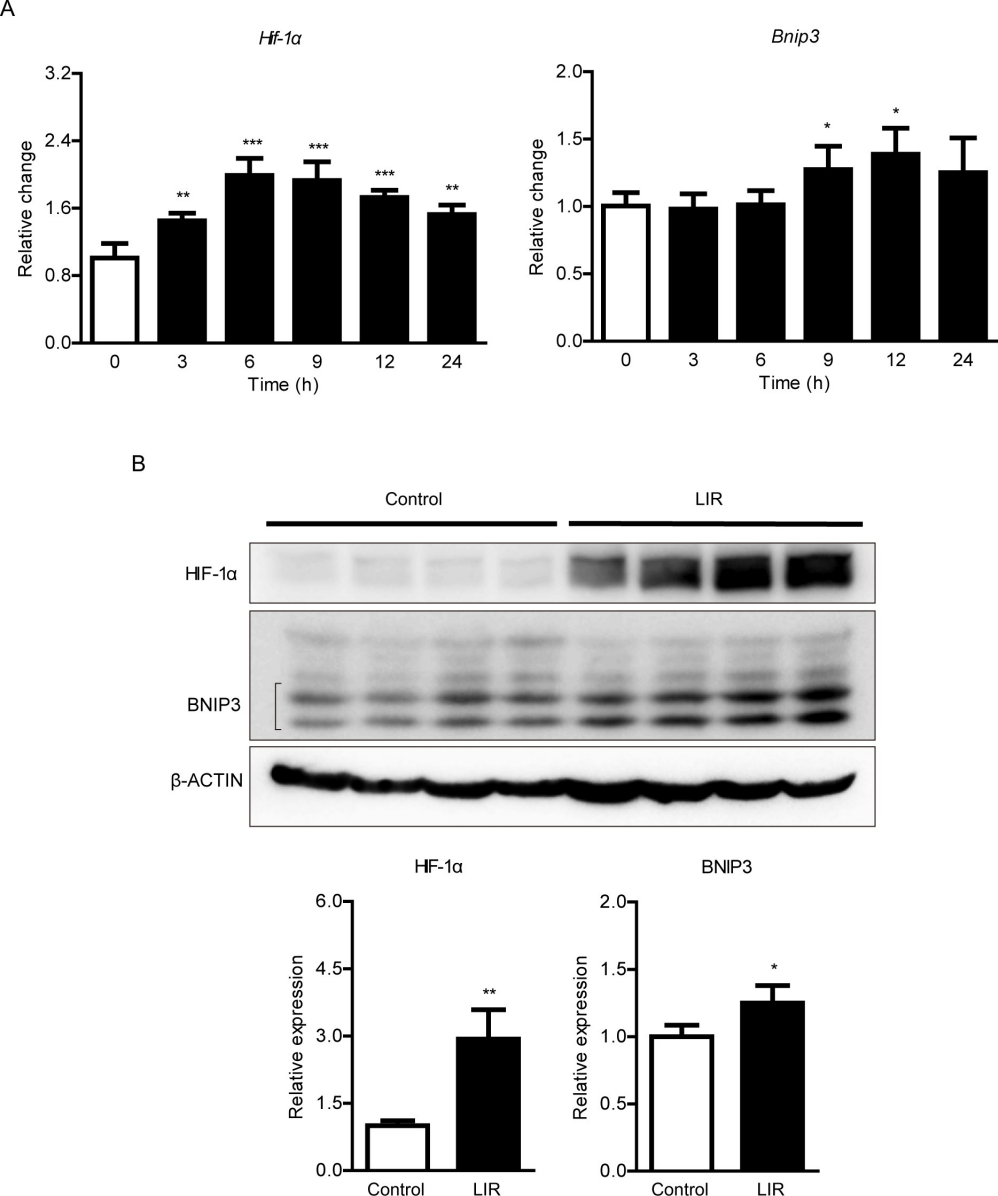
Changes in hypoxia-inducible factor-1α (HIF-1α) and BCL2/adenovirus E1B 19 kDa protein-interacting protein 3 (BNIP3) expressions in a murine model of light-induced retinopathy. (A) Quantitative analyses showed that retinal *Hif-1α* and *Bnip3* mRNA expressions increased after extensive light exposure. Graphs were illustrated as mean ± standard deviation. The data were analyzed using two-way Student’s *t*-test. N = 4 per group. h: hour(s). (B) Images and Quantitative analyses showed that the protein expressions of HIF-1α and BNIP3 (multiple bands in the black parentheses) increased after extensive light exposure. Graphs were shown as mean ± standard deviation. The data were analyzed using two-way Student’s *t*-test. N = 4 per group. LIR: light-induced retinopathy. *P < 0.05, **P < 0.01, ***P < 0.001.

Previous studies demonstrated that HIF-1α expression increased in the retina by various stimuli [18], and its induction could be linked to one of the pathologic processes for disease progression [18, 25]. We examined whether HIF-1α could be induced in our murine model of light-induced retinopathy (Fig 1). The expressions of *Hif-1α* and its downstream gene *Bnip3* increased in the retina after extensive light exposure (Fig 1A). When it comes to *Hif-1α*, its mRNA expression started to increase 3 hours after the exposure. Then, its expression was maintained for 24 hours. *Bnip3* mRNA expression started to increase 9 hours after the exposure and its increase was maintained detected for 12 hours. Next, we examined the protein levels of HIF-1α and BNIP3 in the retina after extensive light exposure (Fig 1B). HIF-1α and BNIP3 expressions significantly increased 24 hours after the exposure.

### A HIF inhibitor halofuginone treatment prevents retinal dysfunction in a murine model of light-induced retinopathy

**Fig 2:**
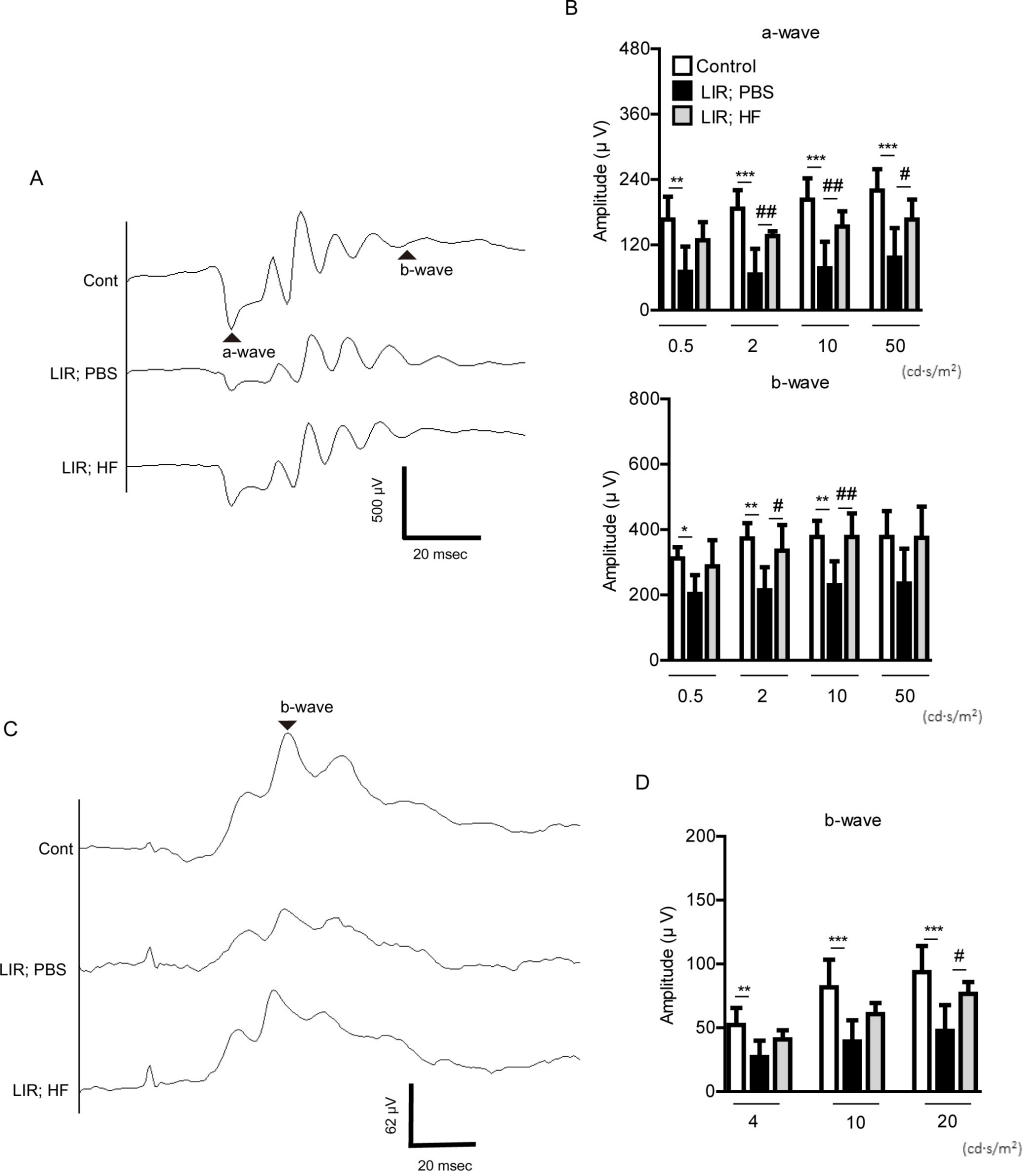
Retinal functional alterations by halofuginone treatment in a murine model of light induced retinopathy. (A and B) Images and quantitative analyses showed that the amplitudes of scotopic a- and b-waves were reduced after extensive light exposure and their reductions were suppressed by halofuginone treatment (flash intensities, 0.5, 2, 10 and 50 cd.s/m^2^). Graphs were shown as mean ± standard deviation. The data were analyzed using one-way ANOVA followed by a Bonferroni post-hoc test. N = 6–7 per group. (C and D) Images and quantitative analyses showed that the amplitude of photopic b-wave was reduced after extensive light exposure and its reductions were suppressed by halofuginone treatment (flash intensities, 4, 10 and 20 cd.s/m^2^). Graphs were shown as mean ± standard deviation. The data were analyzed using one-way ANOVA followed by a Bonferroni post-hoc test. N = 6–7 per group. LIR: light-induced retinopathy. HF: halofuginone. Cont: control (naïve mice). *P < 0.05, **P < 0.01, ***P < 0.001, #P < 0.05, ##P < 0.01. Triangles were used to mark scotopic a-wave and b-wave, and photopic b-wave.

We examined whether a HIF inhibitor halofuginone could show preventive effects on retinal function in our murine model of light-induced retinopathy (Fig 2). The amplitudes of scotopic a-wave and b-wave were significantly reduced 7 days after extensive light exposure (Fig 2A and 2B). We found that halofuginone treatment significantly suppressed their reductions (Fig 2A and 2B). We also found the similar preventive effect of halofuginone under the photopic ERG condition (Fig 2C and 2D).

### A HIF inhibitor halofuginone treatment prevents histopathological retinal damage in a murine model of light-induced retinopathy

**Fig 3:**
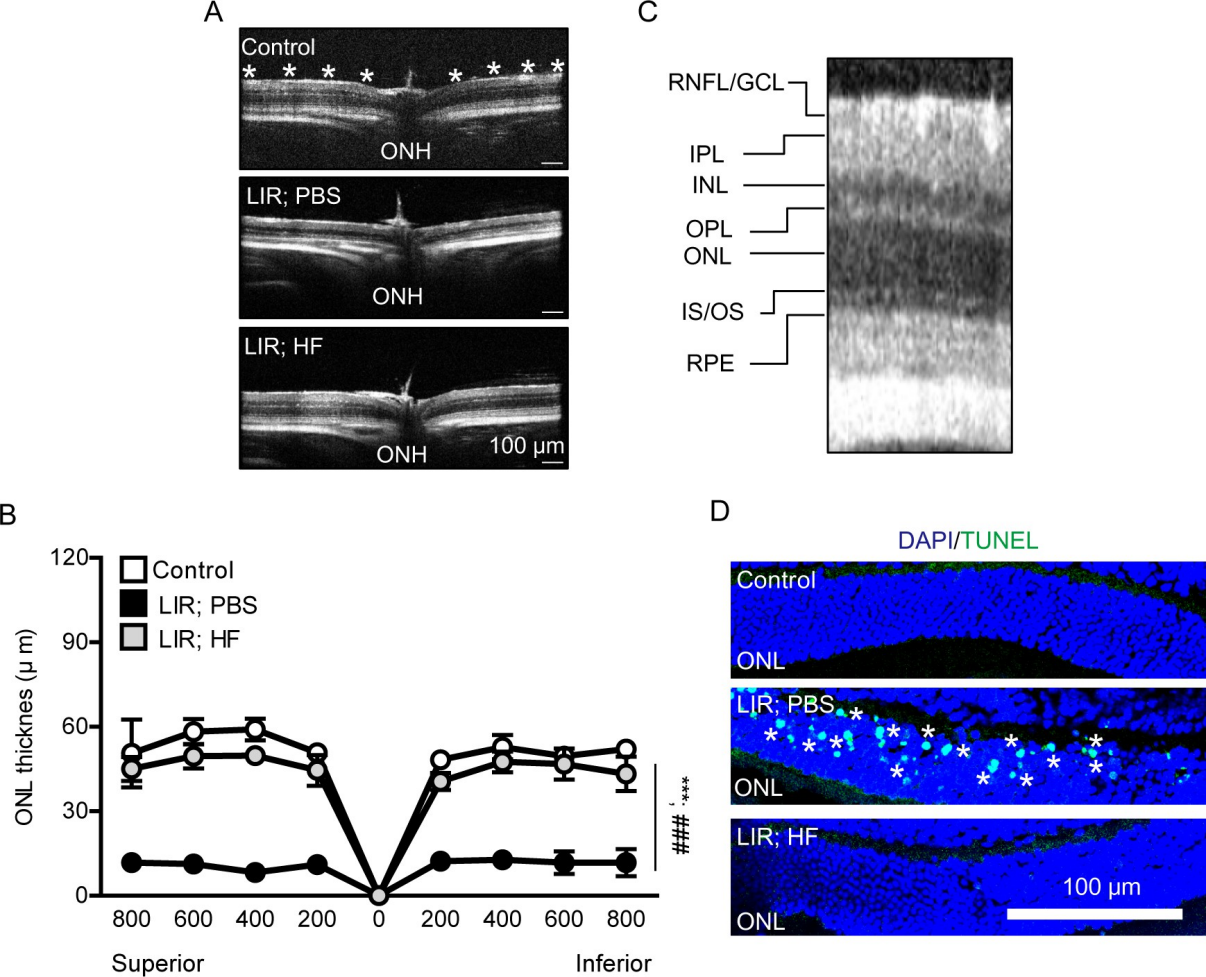
Histological alterations by halofuginone treatment in a murine model of light induced retinopathy. (A and B) Retinal images of the optical coherence tomography (OCT) and quantitative analyses showed that halofuginone treatment suppressed outer retinal thinning in light-induced retinopathy, analyzed at 200, 400, 600, and 800 μm (dot marks were displayed at each point with a 200 μm interval) from optic nerve head (ONH, 0). N = 4 per group. The data were analyzed using two-way ANOVA followed by a Bonferroni post hoc test. Graphs were shown as mean  ±  standard deviation. (C) The enlarged representative retinal image of OCT to detect the outer nuclear layer. RNFL/GCL: the retinal nerve fiber layer and ganglion cell layer. IPL: the inner plexiform layer. INL: the inner nuclear layer. OPL: the outer plexiform layer. ONL: the outer nuclear layer. IS/OS: the junction between the photoreceptor outer and inner segments. RPE: the retinal pigment epithelium. (D) The TUNEL assay image showed that halofuginone treatment protected against outer retinal cell death (marked with white asterisks) in light-induced retinopathy. ONL: the outer nuclear layer. LIR: light-induced retinopathy. HF: halofuginone. ***P < 0.001, ###P < 0.001.

We examined whether a HIF inhibitor halofuginone could show preventive effects on histopathological retinal damage in our murine model of light-induced retinopathy (Fig 3). As murine models of light-induced retinopathy generally show outer retinal thinning [6], we also reproduced this finding in our model 7 days after extensive light exposure (Fig 3A–3C). Under this pathologic condition, halofuginone treatment dramatically suppressed its thinning 7 days after the exposure.

Previous studies demonstrated that light-induced cell death could be detected at the early time point after light exposure in models of light-induced retinal damage [40–43]. Along with this notion, we used the TUNEL assay to examine cell death in the outer retina (Fig 3D). TUNEL-positive cells were detectable in the outer retina 24 hours after extensive light exposure. And its cell death was not induced in the halofuginone-administered retina.

### A HIF inhibitor halofuginone treatment reduces HIF-1α expression in a murine cone photoreceptor cell line

**Fig 4:**
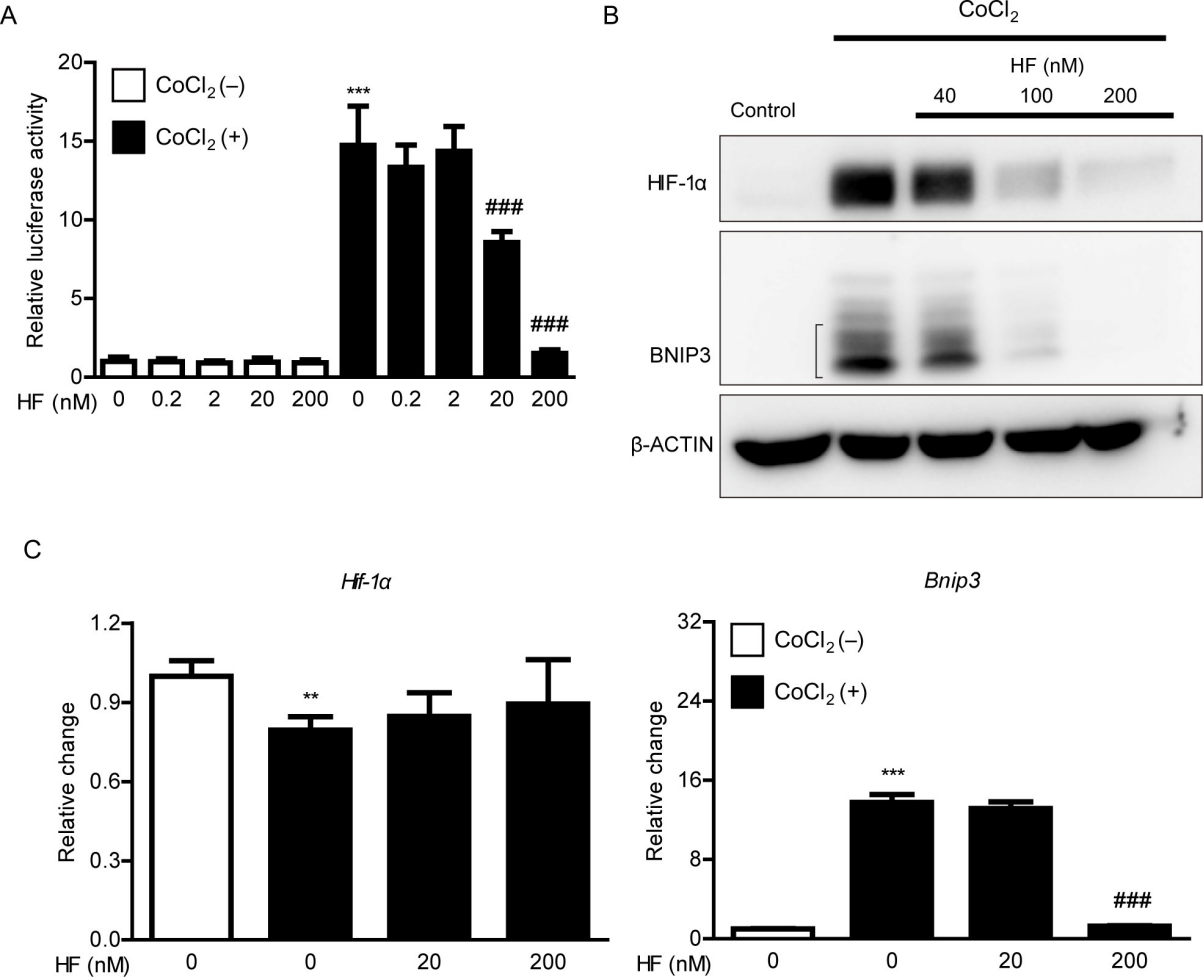
Changes in hypoxia-inducible factor-1α (HIF-1α) and BCL2/adenovirus E1B 19 kDa protein-interacting protein 3 (BNIP3) expressions by halofuginone treatment in a 661W photoreceptor cell line. (A) A quantitative analysis of the HIF-reporter luciferase assay using 661W cells showed that CoCl_2_-induced HIF activity was suppressed by halofuginone treatment. The data were analyzed using one-way ANOVA followed by a Bonferroni post-hoc test. N = 3–9 per group. (B) A western blot image showed that halofuginone treatment reduced CoCl_2_-induced HIF-1α and BNIP3 protein expressions in 661W cells. (C) A quantitative analysis showed that CoCl_2_-induced *Bnip3* mRNA expression was reduced by halofuginone treatment in 661W cells while *Hif-1α* mRNA expression was not changed. The data were analyzed using one-way ANOVA followed by a Bonferroni post-hoc test. N = 3–6 per group. HF: halofuginone. **P < 0.01, ***P < 0.001, ###P < 0.001.

To explain whether a HIF inhibitor halofuginone could show suppressive effects on HIF induction in photoreceptors, we applied 661W cells (a murine photoreceptor cell line) to our system. Previously, we found that CoCl_2_-induced HIF activity could be reduced by halofuginone treatment, analyzed by the luciferase assay [28]. In our current system, we further examined which concentrations of halofuginone could effectively reduce CoCl_2_-induced HIF activity (Fig 4A). Although suppressive effects of halofuginone on HIF activity were well-confirmed, halofuginone had a narrow therapeutic window (20 to 200 nM). 200 nM halofuginone was strongly effective, while 20 nM halofuginone partially reduced CoCl_2_-induced HIF activity. Based on the western blot analysis, the protein expressions of HIF-1α and its downstream gene BNIP3 were dose-dependently reduced by halofuginone treatment (Fig 4B). Under this condition, 200 nM halofuginone was only effective to clearly reduce HIF-1α and BNIP3 expressions. When it comes to qPCR, *Bnip3* mRNA expression was reduced by halofuginone treatment (200 nM), while *Hif-1α* mRNA expression was not changed (Fig 4C). This indicates that halofuginone may suppress HIF-1α stabilization at the protein level rather than at the mRNA level. Furthermore, a high dose of halofuginone might be effective to suppress HIF activity.

## Discussions

The current study demonstrated that retinal HIF expression increased in a mouse model of light-induced retinopathy, and a HIF inhibitor halofuginone could exert preventive effects on outer retinal degeneration in the model. Furthermore, HIF expression could be reduced by halofuginone treatment in 661W under CoCl_2_-induced pseudohypoxic condition. Although halofuginone treatment has been suggested as a promising therapeutic in retinal degeneration (especially, the inner retina) [28], as far as we know, our current data first expanded the preventive role of halofuginone on outer retinal degeneration in a murine model of light-induced retinopathy.

HIF-1α expression increased in the retina after light exposure, and light-induced retinal dysfunction and outer retinal damage were reduced by the treatment of the HIF inhibitor halofuginone. In our previous study, *Hif-1α* knock-out could reduce apoptosis in 661W cells under an oxidative stress condition [29]. Light-induced retinal degeneration has been linked with oxidative stress in the retina [44]. Increases in photoreceptor cell death in human retinal organoids were seen in response to oxidative stress [21]. Under this condition, HIF-1 expression increased in the organoids [21]. During chronic HIF activation, deficits in mitochondrial tricarboxylic acid cycle (TCA cycle) or oxidative phosphorylation (OXPHOS) could be related to the progression of rod photoreceptor degeneration [45]. Even though whether light-induced oxidative stress directly causes increases in HIF-1α expression or vice versa has not been clearly understood, based on our previous and present data, we speculated that the inhibition in HIF-1α expression could decrease cell death in the outer retina or retinal dysfunction. However, a recent study showed that photoreceptor cell death could be promoted by HIF inhibition using digoxin (another HIF inhibitor) treatment in a NaIO_3_-induced mouse model for ocular oxidative stress [21]. Treatment durations, concentrations, or methods of HIF inhibitor drugs are needed to be clearly confirmed, which will be further studied.

BNIP3, one of the mitochondrial proteins, is involved in mitochondrial dysfunctions. BNIP3 has been reported involved in the activation of cell death interacting with BCL-2, BCL-XL, BAK, or BAX [46, 47]. Thereafter, BNIP3 was found as one of the downstream target genes for HIF-1 [29, 48–50]. In this aspect, we previously demonstrated that the HIF-1α/BNIP3 pathway could be one of the promising therapeutic targets in retinal degeneration in a murine model of retinal ischemia/reperfusion injury and unilateral common carotid artery occlusion, or a 661W photoreceptor cell line [28, 29, 51]. *Hif-1α* or *Bnip3* knock-out reduced apoptosis in 661W cells under an HIF-induced oxidative stress condition. In our current study, halofuginone treatment suppressed the expressions in HIF-1α as well as BNIP3 in 661W cells. Furthermore, we found that pathologic HIF-1α and BNIP3 expressions in the retina increased by extensive light exposure. Taken together, although more studies are needed, the HIF-1α/BNIP3 pathway could be considered as a therapeutic target in outer retinal degeneration.

Halofuginone has been reported to be effective against a variety of diseases, including parasitic diseases, fibrosis, cancer, and autoimmune diseases in many animal species [52, 53]. There have also been reports of its use in animals [54]. Although repositioning halofuginone therapy to the treatment for photoreceptor cell death in animals or humans needs more considerations in terms of the drug dosage (a range of its effective concentration to suppress HIF activity was narrow) and administration time-points and methods, the further study with halofuginone therapy may reduce barriers to the current clinical therapy limits.

In summary, we preliminarily attempted to apply a promising halofuginone therapy to a murine model of light-induced retinopathy. As the outcomes, retinal functional deficits and outer retinal thinning were suppressed by halofuginone treatment. Furthermore, pathologic HIF-1α and cell death-related BNIP3 inductions could be directly reduced by halofuginone treatment in photoreceptor cells. Although more understandings on the mechanism of action of the HIF inhibition therapy in outer retinal degeneration are required, we shortly suggest that halofuginone can be one of the promising HIF inhibiting therapeutics for preventing the progression of outer retinal cell death-related ocular diseases.

## Supporting information

S1 Raw images Uncropped western blot images are attached in this file. (PDF)

S1 Appendix Datasets are attached in this file. (XLSX)
