# Supplementary figures and images for: Halofuginone prevents outer retinal degeneration in a mouse model of light-induced retinopathy
Source: PLoS One. 2024 Mar 27;19(3):e0300045. doi: 10.1371/journal.pone.0300045 (PMC10971573; doi:10.1371/journal.pone.0300045)

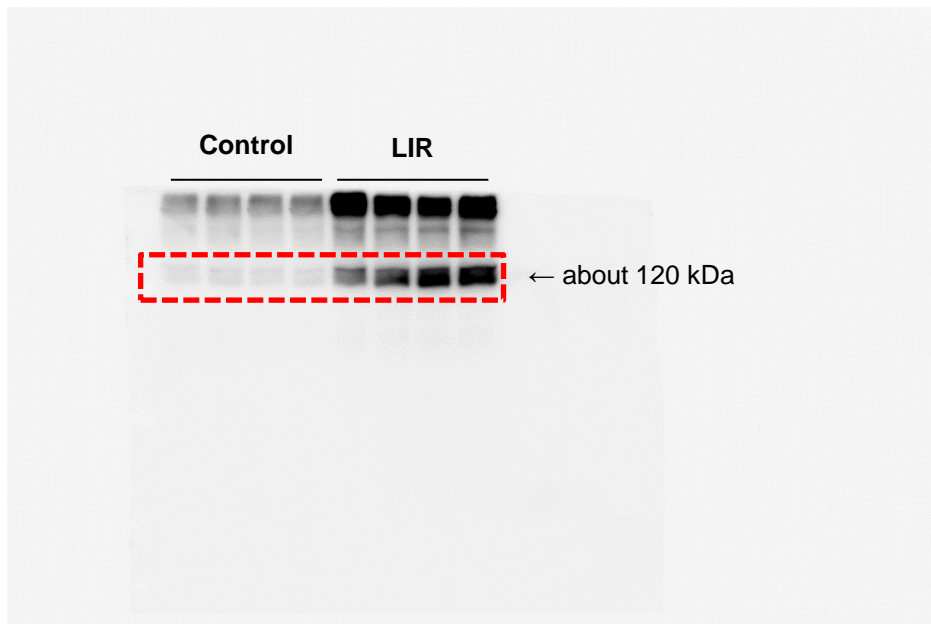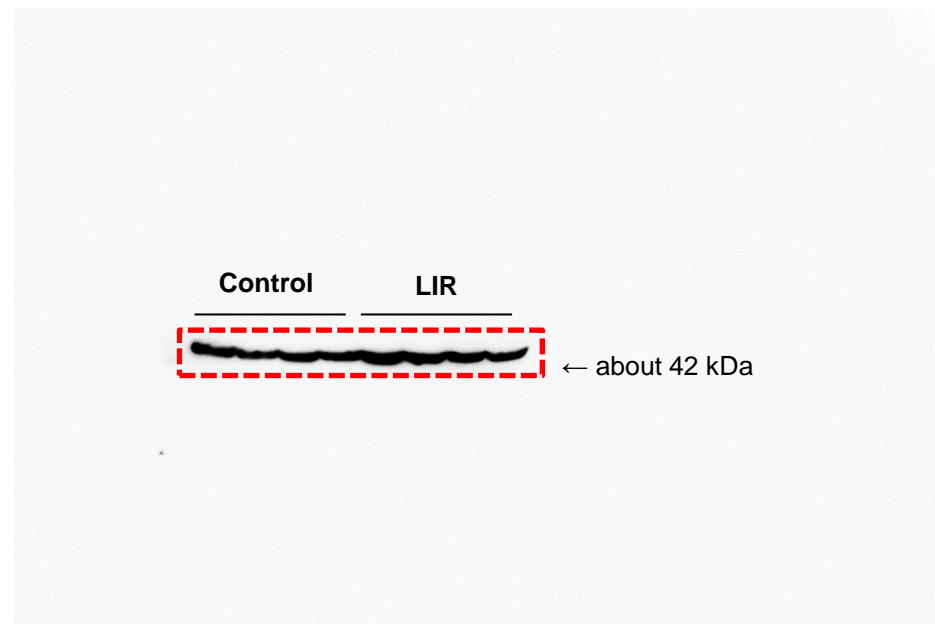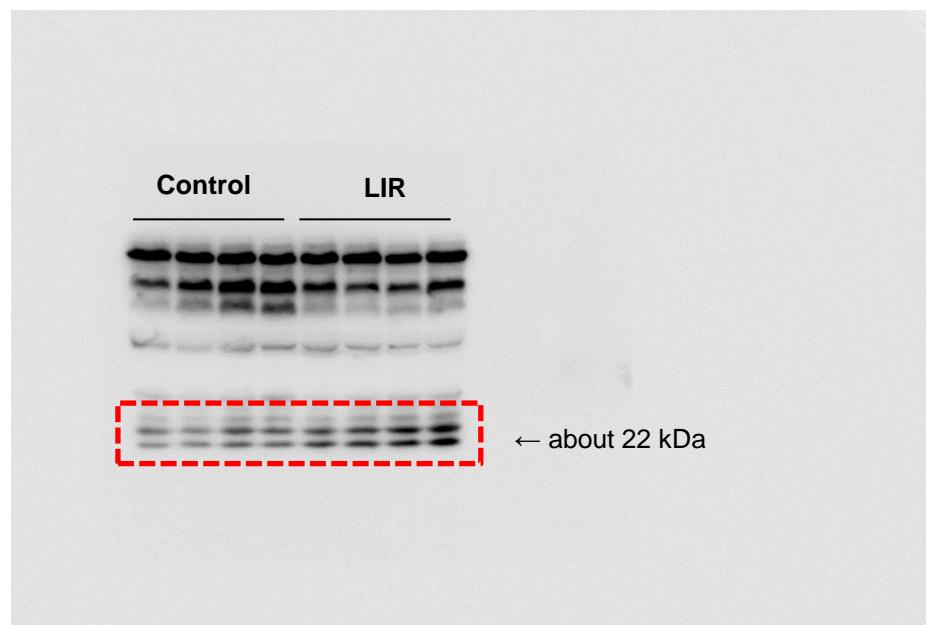

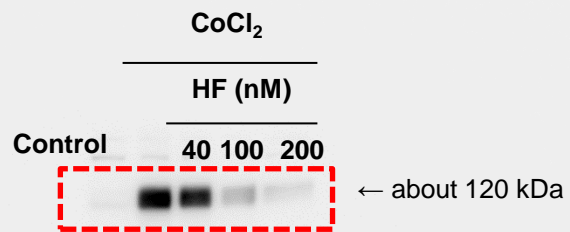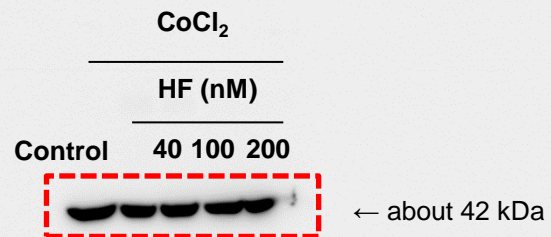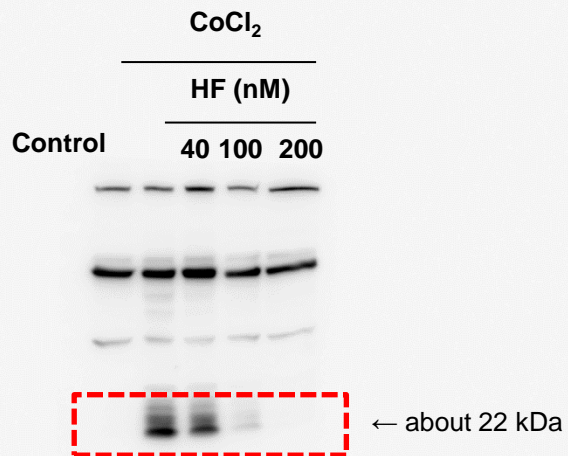

Supplement: S1 Raw images — (PDF) [file pone.0300045.s001.pdf]
